# Quarterly Report of the House Committee

**Published:** 1917-06-09

**Authors:** 


### THE LONDON HOSPITAL.

### Quarterly Report of the House Committee.

STAFF difficulties continue, although the Director-General of the Army Medical Service has arranged to allow students to remain after qualification for three months before joining up, an arrangement which is of service not only to the hospital, but obviously to the Army, as they gain three months' experience in the wards before going out to work in France or wherever they are needed most to look after the needs of the Army. The American doctors, who have so kindly been giving their help, are mostly still at the hospital, but their future is uncertain; one or two of them have left to return to America, as they feel that it is now their duty to join the American Army; while, on the other hand, it has been made possible for American doctors to join the English Royal Army Medical Corps.

THE SYPHILIS DEPARTMENT.—An interesting report has been prepared by Dr. Sequeira, who is in charge of the syphilis wards and syphilis out-patient department, on the first year's work of this department. 620 patients have been treated during the twelve months—293 males and 327 females. These include about thirty children under sixteen years of age, and five babies under one year. 1,616 injections of salvarsan have been given, and all cases have been verified by the Wassermann test, and the progress of the patient watched by examinations of the blood at intervals. Special efforts have been made to trace "contacts"—*e.g.*, if a married woman comes for treatment, the children are invited to attend, or the husband. The wards are working in close connection with the maternity department of the hospital, and also, through Dr. Eardley Holland, with certain lying-in charities, admitting women for treatment who had had still-births due to syphilis, or in whom infection had occurred during pregnancy. The value of this work in increasing the birth-rate, and especially in the birth of healthy babies, is obvious, and the results have been most gratifying. Great care is taken with regard to the teaching in the department. Instruction is given regularly in the technique of blood examination and in the modern methods of treatment, and doctors are availing themselves fully of the opportunities offered. The Local Government Board are sending medical officers of health and others working in this branch of preventive medicine from all parts of the country. All patients are provided with printed papers setting forth the dangers and risks of conveying infection. These leaflets are printed both in English and Yiddish.

ALEXANDRA HOME AND HONOUR TO MATRON.—This Nursing Home Extension was, as the governors will have read, formally opened by our President, Her Majesty Queen Alexandra, on the occasion of her visit a week or two ago, the special object of which was to honour the matron by decorating her with the First Class of the Royal Red Cross, which had been bestowed upon her by His Majesty the King as a mark of his appreciation of her great services to the nursing profession and the London Hospital over a period of thirty-six years. Her Majesty most kindly visited the hospital in order to hand over the decoration herself, a fact which is doubtless a source of satisfaction and pride to the governors.

DEATH OF MR. LEOPOLD DE ROTHSCHILD.—It is with great regret that the death of Mr. Leopold de Rothschild is recorded. He was Vice-President of the hospital for forty-four years, and took a great personal interest in the institution, not only subscribing largely to the funds by yearly contributions, but also paying the Marie Celeste Samaritan Society for the convalescence of Jewish patients, and sending weekly contributions of flowers and fruit from his country place for the use of the patients.

WAR ALLOWANCES.—The hospital is paying allowances to members of the lay staff who have joined the Army amounting to over £3,000 a year. A small sub-committee was appointed by the house committee to inquire into the matter, and a scheme was formulated under which no man is allowed to increase his income by joining the Army, his income being made up to what he was earning at the hospital, except in the case of men who are unmarried and have no dependents.

DISPENSARY.—An interesting report has been submitted to the house committee by the dispensary sub-committee. Mr. Hocking, the dispenser, pointed out that in his comparatively small department ten men had joined the Army. He drew attention to the fact that the engagement of lady dispensers had been a great success. The net cost of the department showed an increase of £968. This increase was entirely owing to drugs. With regard to dressings, there was a decrease of £335. Mr. Hocking gave some interesting examples of increases in cost, of which methylated spirit was one. This cost £218 more, in spite of the fact that 460 gallons less were used. Mr. Hocking also gave some instances of avoided large expenditure owing to large stocks of certain drugs being held at the beginning of the war. Potassium bromide, for instance, which is used in considerable quantities, especially in the out-patient department, in epileptic cases, had risen to the enormous price of 25s. per lb., yet throughout the war stock costing 1s. 6½d. per lb. was used. The greatest credit is due to Mr. Hocking for his continued excellent management of the department and for his foresight.

